# Cytotoxic Steroidal Saponins from the Flowers of *Allium leucanthum*

**DOI:** 10.3390/molecules13122925

**Published:** 2008-11-26

**Authors:** Lasha Mskhiladze, Jean Legault, Serge Lavoie, Vakhtang Mshvildadze, Jumber Kuchukhidze, Riad Elias, André Pichette

**Affiliations:** 1 Tbilisi State Medical University, Departement of Pharmacognosy, 33, Vazha Pshavela Ave., Tbilisi, 0177, Georgia; E-mail: lasha@tsmu.edu (L. M.); 2 Laboratoire de Pharmacognosie, Faculté de Pharmacie, Université de la Méditerranée; 27 Boul. Jean Moulin, 13385 Marseille, cedex 5, France; E-mail : riad.elias@pharmacie.univ-mrs.fr (R. E.); 3 Laboratoire LASEVE, Université du Québec à Chicoutimi, 555 Boul. de l’Université, Département des Sciences Fondamentales, Chicoutimi, Québec, Canada; E-mails : jean_legault@uqac.ca (J. L.), serge_lavoie@uqac.ca (S. L.), vakhtang_mshvildadze@uqac.ca (V. M.)

**Keywords:** *Allium leucathum*, Spirostane saponins, Structure determination, Cytotoxic activity.

## Abstract

*Allium leucanthum* C. Koch is an endemic Caucasian species that grows in Georgia. The flowers are used in traditional medicine. Phytochemical investigation allowed the isolation of seven spirostanol type saponins from the flowers. Their structures were elucidated on the base of NMR and HRESIMS spectrometry data. A new compound, which we have named leucospiroside A (**5**), has been identified as (25*R*)-5α-spirostane-2α,3β,6β-triol 3-O-β-glucopyranosyl-(1→3)-β-glucopyranosyl-(1→2)-[β-glucopyranosyl-(1→3)]-β-glucopyranosyl-(1→4)-β-galactopyranoside. The six others were known substances, but are described in this plant for the first time. The crude extract, spirostanol and furostanol fractions, as well as isolated compounds, were evaluated for their *in vitro* cytotoxic activity. Compounds **1**-**3** and **5** were found to be the most active, with relatively similar IC_50_ values ranging from 3.7 to 5.8 µM for a lung cancer cell line (A549) and 5.6 to 8.2 µM for a colon cancer cell line (DLD-1).

## Introduction

Genus *Allium* includes up to 500 species in the world flora. Among them, some 70 grow in the Caucasian region and 35 species are described in Georgia [1]. *Allium leucanthum* C. Koch (Alliaceae), called “whiteflower onion” in Georgia, is a Caucasian endemic species [2] and, along with other *Allium* species, is widely used in Georgian traditional medicine as an antifungal, antiseptic and antibacterial remedy [3,4]. Various secondary metabolites were identified in genus *Allium* [5]. Among them, steroidal saponins have been investigated for their antibacterial [6], antifungal [7,8] and antioxidant [9,10] activities. Furthermore, steroidal saponins isolated from different species of onions showed a significant cytotoxic activity against murine fibrosarcoma [11], lung carcinoma [12], human melanoma [13] and human leukemia [14].

**Figure 1:**
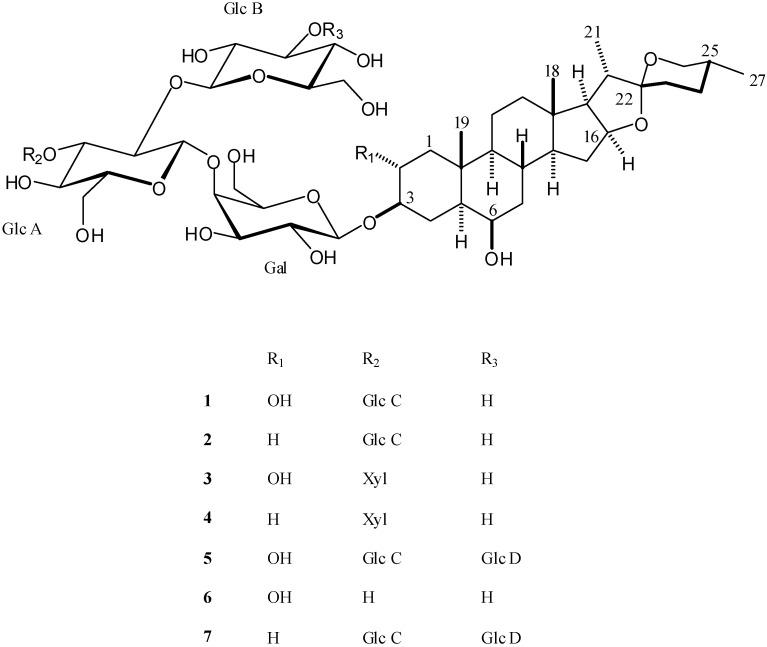
The structures of the steroidal saponins isolated from *A. leucanthum*.

Steroidal saponins of only one species, *A. erubescens*, which grows in Georgia, have been reported [15]. This prompted us to undertake the phytochemical and cytotoxic studies of different extracts prepared from *Allium leucanthum* flowers. This bioassay-guided isolation allowed us to obtain seven cytotoxic spirostanol saponins (**1**-**7**, Figure 1).

This paper describes the isolation of several spirostanol saponins from the flowers of *Allium leucanthum* C. Koch, and the structural determination of new compound **5** by HRESIMS, ^1^H and ^13^C NMR spectroscopy. Furthermore, the *in vitro* cytotoxic activities of saponins and related aglycons are reported.

## Results and Discussion

The flowers of *Allium leucanthum* C. Koch were extracted with aqueous methanol. The cytotoxicity of the extract was evaluated against human tumor and healthy cell lines including lung adenocarcinoma (A549), colorectal adenocarcinoma (DLD-1) and normal skin fibroblasts (WS1) (Table 1). The methanolic extract was shown to strongly inhibit the growth of tumor cell lines, with an IC_50_ of 15 ± 3 µg/mL for A549 and 19.6 ± 0.9 µg/mL for DLD-1. However, the extract was not found to be selective toward tumor cell lines when compared with healthy cell line WS1 (IC_50_, 10.6 ± 0.8 µg/mL). The methanolic extract was then partitioned between water and 1-butanol to eliminate hydrophylic compounds. The 1-butanol extract was then chromatographied on Diaion resin under gradient conditions with MeOH-H_2_O (0→100%). Two fractions were obtained and were shown to contain spirostanol and furostanol saponins. The cytotoxicity of both fractions was tested against tumor and normal cell lines. The spirostanol fraction was more active than the methanolic extract. In contrast, the furostanol fraction was inactive against both tumor cell lines (IC_50_ > 90 µg/mL). Therefore, the chemical composition of the spirostanol fraction was studied and seven compounds **1**-**7** were isolated using open column chromatography.

**Table 1:** Cytotoxicity of extract and saponin fractions from *A. leucanthum.*

Extract and fractions	IC_50_ (μg/ml ± SD)^a^		
	A549	DLD-1	WS1
Methanol	15 ± 3	19.6 ± 0.9	10.6 ± 0.8
Spirostanol	8.8 ± 0.4	11.1 ± 0.6	6.3 ± 0.3
Furostanol	92 ± 19	> 200	32 ± 2

^a^ Data represent mean values (± standard deviation) for three independent assays.

The structures of the known compounds (**1**-**4** and **7**) were determined by comparison of the ^1^H- and ^13^C-NMR spectral data with those reported in the literature. They were identified for the first time in *Allium leucanthum* as yayoisaponin C (**1**) [16], eruboside B (**2**) [7, 15], aginoside (**3**) [17, 18], (25*R*)-5α-spirostane-3β,6β-diol 3-O-β-d-glucopyranosyl-(1→2)-[β-d-xylopyranosyl-(1→3)]-β-d-glucopyranosyl-(1→4)-β-d-galactopyranoside (**4**) [19] and (25*R*)-5α-spirostane-3β,6β-diol 3-O-β-d-glucopyranosyl-(1→3)-β-d-glucopyranosyl-(1→2)-[β-d-glucopyranosyl-(1→3)]-β-d-glucopyranosyl-(1→4)-β-d-galactopyranoside (**7**) [20]. Compound **6**, namely (25*R*)-5α-spirostane-2α,3β,6β-triol 3-O-β-d-glucopyranosyl-(1→2)-β-d-glucopyranosyl-(1→4)-β-d-galactopyranoside, was already reported in the literature [17], but since NMR data were missing, they are reported in this paper (Table 3).

Compound **5**, [α]_D_^25^ = -51.9° (c 0.3, CHCl_3_/MeOH/H_2_O), was obtained as a white amorphous solid, and its molecular formula was suggested to be C_57_H_94_O_30_ by the HRESIMS data [*m*/*z* 1281.5715, (M+Na)^+^, Δ -0.7 mmu]. The ^1^H-NMR spectrum of **5** (Table 2) showed characteristic proton signals due to two tertiary methyls at δ_H_ 0.82 (3H, s) and δ_H_ 1.24 (3H, s), two secondary methyls at δ_H_ 0.69 (3H, d, *J* = 5.3 Hz) and δ_H_ 1.15 (3H, d, *J* = 6.9 Hz) and five anomeric protons at δ_H_ 5.00 (1H, d, *J* = 7.8 Hz), δ_H_ 5.10 (1H, d, *J* = 7.8 Hz), δ_H_ 5.17 (1H, d, *J* = 7.8 Hz), δ_H_ 5.22 (1H, d, *J* = 7.8 Hz) and δ_H_ 5.64 (1H, d, *J* = 7.9 Hz). The ^13^C NMR spectrum showed 57 peaks: 30 for the sugar moieties including five anomeric carbons at δ_C_ 105.0, 104.0, 103.9, 103.5 and 102.7, and 27 for the aglycones part. The latter were found very similar to those of **1**, suggesting that the aglycone of **5** was also agigenin.

The NMR chemical shifts for galactose and glucose A-C, as determined from HSQC and multiple 1D-selective TOCSY experiments, were very similar to those reported for **1** whereas C-3 of glucose B of **5** (δ_C_ 87.6) was significantly shifted downfield by 9.8 ppm from that of **1** (δ_C_ 77.8), indicating that another sugar unit was attached to C-3 of glucose B. The same sugar units were also identical to those of yayoisaponin A [16] except for xylose which was identified as glucose (glucose D) in compound **5** based on the ^13^C-NMR chemical shift [21]. Indeed, the identification of the sugar units was confirmed by acid hydrolysis of compound **5** where only glucose and galactose could be detected by TLC analysis. These sugar linkages were confirmed by long range correlations on the HMBC spectrum between H-1 of Gal (δ_H_ 5.00) and C-3 of aglycone (δ_C_ 84.2), H-1 of Glc A (δ_H_ 5.10) and C-4 of Gal (δ_C_ 79.5), H-1 of Glc B (δ_H_ 5.64) and C-2 of Glc A (δ_C_ 80.4), H-1 of Glc-C (δ_H_ 5.22) and C-3 of Glc A (δ_C_ 88.0) and H-1 of Glc D (δ_H_ 5.17) and C-3 of Glc-B (δ_C_ 87.6). Finally, the anomeric configuration was determined from the large coupling constant of the anomeric protons which range from 7.8 to 7.9 Hz for all the sugar units. Thus, saponin **5** was identified for the first time as (25R)-5α-spirostane-2α,3β,6β-triol 3-O-β-glucopyranosyl-(1→3)-β-glucopyranosyl-(1→2)-[β-glucopyranosyl-(1→3)]-β-glucopyranosyl-(1→4)-β-galactopyranoside and named leucospiranoside A.

The cytotoxic activities of glycosides **1**-**7** were evaluated against A549, DLD-1 and WS1. Etoposide and 5-fluorouracil (5-FU) were used as positive controls. Results presented in Table 4 show that compounds **1**-**3** and **5** possess a relatively similar cytotoxicity against both tumor cell lines, with IC_50_ values ranging from 3.7 to 5.8 µM for A549 and 5.6 to 8.2 µM for DLD-1. The cytotoxic activities of compounds **1**-**3** were previously reported in the literature [16, 22]. When compared with their cytotoxic activities against healthy cell line WS1, the glycosides were not found to be selectively toxic towards the cancer cell lines. The structure-activity relationship shows that the presence of glucose or xylose at position R_2_ of compounds **1** and **3** is important for bioactivity. Indeed, the cytotoxicities of compound **1** and **3** are significantly (p<0.05, ANOVA one way analysis) increased by about three to six times when compared with compound **6** which has neither glucose or xylose at position R_2_. However, the addition of a second glucose at position R_3_ (compound **5**) does not significantly improve cytotoxic activity when compared with compound **1** (p<0.05). The change from a glucose (compound **1**) to a xylose at position R_2_ (compound **3**) with a hydroxyl at position R_1_ does not modify the cytotoxicity of the molecule. However, the absence of a hydroxyl at position R_1_ slightly reduces the activity.

**Table 2:** NMR spectroscopic data of compound **5**^a^.

Aglycone			Sugar moities		
Position	δ_C_^b^	δ_H_^c^	Position	δ_C_^b^	δ_H_^c^
1	46.9 t	2.20 m	Galactose		
		1.25 m	1	102.7 d	5.00 d (7.8)
2	70.5 d	4.11 m	2	72.4 d	4.53 m
3	84.2 d	4.06 m	3	75.1 d	4.18 m
4	31.5 t	2.39 q-like (12.6)	4	79.5 d	4.56 m
		2.16 m	5	75.5 d	4.11 m
5	47.6 d	1.15 m	6	60.9 d	4.54 m, 4.30 m
6	69.9 d	4.01 m	Glucose A		
7	40.4 t	2.05 m	1	103.9 d	5.10 d (7.8)
		1.16 m	2	80.4 d	4.26 m
8	31.9 d	2.11 m	3	88.0 d	4.13 m
9	54.3 d	0.71 m	4	70.2 d	3.80 m
10	36.9 s	-	5	77.2 d	3.82 m
11	21.3 t	1.52 m	6	62.5 d	4.43 m, 4.00 m
		1.37 m	Glucose B		
12	40.0 t	1.67 m	1	103.5 d	5.64 d (7.9)
		1.08 m	2	74.4 d	4.09 m
13	40.8 s	-	3	87.6 d	4.24 m
14	56.1 d	1.13 m	4	69.5 d	3.92 m
15	32.1 t	1.46 m	5	77.5 d	3.89 m
		1.23 m	6	62.1 d	4.38 m, 4.29 m
16	81.2 d	4.58 m	Glucose C		
17	62.8 d	1.85 t (7.3)	1	104.0 d	5.22 d (7.8)
18	16.6 q	0.82 s	2	75.1 d	4.02 m
19	17.0 q	1.24 s	3	77.9 d	4.21 m
20	42.0 d	1.94 quint. (6.7)	4	71.3 d	4.07 m
21	15.0 q	1.15 d (6.9)	5	78.3 d	4.01 m
22	109.4 s	-	6	62.1 d	4.54 m, 4.18 m
23	31.7 t	1.66 m	Glucose D		
24	29.1 t	1.57 m	1	105.0 d	5.17 d (7.8)
25	30.5 d	1.58 m	2	75.3 d	4.03 m
26	66.9 t	3.62 m	3	77.6 d	4.19 m
		3.51 m	4	71.3 d	4.07 m
27	17.3 q	0.69 d (5.3)	5	78.1 d	3.93 m
			6	62.2 d	4.50 m, 4.17 m

^a^ Spectra recorded at 400.13 MHz (^1^H) and 100.61 MHz (^13^C) in pyridine‑d_5_/D_2_O 10:1. ^b^ Multiplicities of the carbon signals were determined by DEPT experiments. ^c^ Values in parentheses indicate coupling constant (*J* in Hz).

**Table 3:** NMR spectroscopic data of compound **6.**

Aglycone			Sugar moities		
Position	δ_C_^b^	δ_H_^c^	Position	δ_C_^b^	δ_H_^c^
1	47.3 t	1.90 m	Galactose		
		0.92 m	1	102.9 d	4.39 d (7.8)
2	71.4 d	3.66 m	2	72.9 d	3.66 m
3	85.3 d	3.48 m	3	75.6 d	3.52 m
4	31.6 t	1.83 m	4	80.4 d	4.02 br d (3.2)
		1.76 m	5	75.5 d	3.52 m
5	48.5 d	1.20 m	6	61.2 d	3.89 m
6	71.6 d	3.80 m			3.65 m
7	40.8 t	1.83 m	Glucose A		
		1.18 m	1	104.8 d	4.56 d (7.8)
8	30.8 d	1.96 m	2	84.8 d	3.48 m
9	55.6 d	0.78 m	3	78.2 d	3.56 m
10	37.9 s	-	4	71.7 d	3.22 m
11	22.2 t	1.56 m	5	78.0 d	3.29 m
		1.44 m	6	63.2 d	3.88 m
12	41.1 t	1.76 m			3.58 m
		1.18 m	Glucose B		
13	41.8 s	-	1	106.1 d	4.67 d (7.6)
14	57.2 d	1.17 m	2	76.2 d	3.27 m
15	32.8 t	2.00 m	3	77.7 d	3.37 m
		1.29 m	4	70.9 d	3.37 m
16	82.2 d	4.39 m	5	78.7 d	3.35 m
17	63.9 d	1.75 m	6	62.2 d	3.97 m
18	17.0 q	0.82 s			3.80 m
19	17.3 q	1.06 s			
20	43.0 d	1.90 m			
21	14.9 q	0.96 d (7.0)			
22	110.6 s	-			
23	32.5 t	1.70 m			
		1.56 m			
24	29.9 t	1.62 m			
		1.42 m			
25	31.5 d	1.59 m			
26	67.9 t	3.44 m			
		3.32 m			
27	17.5 q	0.79 d (6.4)			

^a^ Spectra recorded at 400.13 MHz (^1^H) and 100.61 MHz (^13^C) in CD_3_OD. ^b^ Multiplicities of the carbon signals were determined by DEPT experiments. ^c^ Values in parentheses indicate coupling constant (*J* in Hz).

**Table 4:** Cytotoxicity of saponins and genins from *A. leucanthum.*

Compounds	IC_50_ (μM ± SD)		
	A549	DLD-1	WS1
Yayoisaponin C (**1**)	3.7 ± 0.7	5.6 ± 0.2	3.0 ± 0.2
Eruboside B (**2**)	5.3 ± 0.5	8.2 ± 0.4	3.6 ± 0.2
Aginoside (**3**)	5.8 ± 0.9	7.9 ± 0.5	3.6 ± 0.2
Compound **4**	9 ± 1	13 ± 1	3.1 ± 0.1
Leucospiroside A (**5**)	5.0 ± 0.1	7.2 ± 0.1	4.55 ± 0.07
Compound **6**	22 ± 2	22 ± 2	14.5 ± 0.5
Compound **7**	7.8 ± 0.4	8.9 ± 0.2	7.7 ± 0.2
Etoposide	1.1 ± 0.1	4.8 ± 0.8	n.d.
5-fluorouracil	48 ± 18	11 ± 2	20 ± 2

n.d.: not determined

In conclusion, the steroidal saponin leucospiroside A (**5**) reported for the first time, was isolated from *Allium leucanthum* along with six other saponins (**1**-**4** and **6**-**7**) which were described for the first time in this endemic species. Compounds **1**-**3** and **5** were found to be the most active compounds. For all steroidal saponins, the presence of sugar moieties is important for the activity.

## Experimental Section

### General experimental

IR spectra were recorded (neat) using a Perkin-Elmer Spectrum One spectrometer. ^1^H- and ^13^C- NMR chemical shifts in ppm were referenced with the residual solvent (CD_3_OD) signals (δ_H_ 3.31 and δ_C_ 49.0) or with TMS as internal standard (for pyridine-*d*_5_/H_2_O). Spectra were recorded on a Bruker DRX-500 and a Bruker Avance 400 instrument. High resolution electrospray ionization mass spectrum was conducted in positive mode on an Applied Biosystems/MDS Sciex QSTAR XL QqTOF MS system. Optical rotation [α]_D_^25^ was measured on an Autopol IV polarimeter. For column chromatography, silica gel 60 (40-63 µm, Merck) and Diaion HP20 resin (Mitsubishi) were used. TLC analysis of saponins were performed on Silica gel 60 F_254_ plates (Merck) and eluted with CH_2_Cl_2_-MeOH-H_2_O (26:14:3). Spots were detected by spraying the plates with vanillin-sulphuric acid (in EtOH) reagent, followed by heating at 110°C (spirostanols were coloured in yellow and furostanols in dark green color).

### Plant material

The flowers of *Allium leucanthum* C. Koch were collected in the Dmanisi region of Georgia (June 2005) and identified by Dr Jumber Kuchukhidze. The flowers were dried by microwave irradiation (Pr KS–22E, 850 W, 2450 MHz) to avoid the enzymatic hydrolysis of bidesmosides (saponins in raw and dry material were evaluated by TLC). A vouched specimen is kept in the department of Pharmacognosy, Faculty of pharmacy, Tbilisi, Georgia (flowers N° AL 0605).

### Extraction and isolation

Dried and powdered flowers of *Allium leucanthum* (500 g) were extracted with MeOH-H_2_O (8:2 v/v, 3 L). After evaporation of the solvent, the residue (79 g) was suspended in water and the saponins were extracted with *n*-BuOH. The *n*-BuOH extract (32 g) was chromatographied over Diaion HP 20, using MeOH-H_2_O as eluent in gradient conditions (0→100%). The spirostanol fraction (14.5 g) was collected in MeOH-H_2_O (7:3, v/v) and the furostanol fraction (5.9 g) in MeOH-H_2_O (5:5, v/v). The spirostanol saponins were subjected to CC on silica gel and eluted with CH_2_Cl_2_-MeOH–H_2_O (45:14:2, v/v/v) to give: **1** (236 mg, R_f_ = 0.41), **2** (28 mg, R_f_ = 0.46), **3** (44 mg, R_f_ = 0.51), **4** (15 mg, R_f_ = 0.54), **5** (18 mg, R_f_ = 0.35), **6** (15 mg, R_f_ = 0.56) and **7** (15 mg, R_f_ = 0.37). The purity of the compounds was evaluated by TLC in different solvent systems (showed only one spot on silica gel plates) and ^1^H- and ^13^C-NMR spectral analyses.

### Spectroscopic data for leucospiroside A (**5**)

White amorphous solid. [α]_D_^25^-51.9° (c=0.3, CHCl_3_/MeOH/H_2_O 26:14:3). IR ν_max_: 3373, 2927, 1637, 1452, 1377, 1155, 1073 and 897 cm^-1^; HRESIMS m/z: 1281.5715. Calcd for C_57_H_94_O_30_Na: 1281.5722. ^1^H and ^13^C-NMR data, see Table 2.

### Acid hydrolysis of leucospiroside A (**5**)

A solution of compound **5** (2 mg) in HCl 5% (3 mL) was heated at 100 °C for 4 h. The reaction mixture was treated with chloroform and the aq. phase was neutralized with *N*,*N*-dioctylmethylamine (10% in CHCl_3_) and dried. The monosaccharide part was dissolved in MeOH 50% (1 mL), and analyzed by TLC with reference galactose (R_f_ = 0.33) and glucose (R_f_ = 0.35). TLC identification of monosaccharides was carried out in a CH_2_Cl_2_-CH_3_OH-H_2_O (50:25:5) solvent system, and further developed using an orthophosphoric acid soln. of naphtorezorsinol 5% in EtOH, followed by heating at 110 °C.

### Cell culture

Lung carcinoma (A549), colon adenocarcinoma (DLD-1) and normal skin fibroblast (WS1) human cell lines were purchased from the American Type Culture Collection (ATCC). All cell lines were cultured in minimum essential medium containing Earle’s salts and L-glutamine (Mediatech Cellgro, VA), to which 10% fetal bovine serum (Hyclone), vitamins (1X), penicillin (100 I.U./mL), streptomycin (100 μg/ml), essential amino acids (1X) and sodium pyruvate (1X) (Mediatech Cellgro, VA) were added. Cells were kept at 37°C in a humidified environment containing 5% CO_2_.

### Cytotoxicity assay

Exponentially growing cells were plated at a density of 5 × 10^3^ cells per well in 96-well microplates (Costar, Corning inc.) in 100 µl of culture medium and were allowed to adhere for 16 hours before treatment. Then, 100 µl of increasing concentrations of extract or pure compounds dissolved in culture medium and DMSO (Sigma-Aldrich) were added. The final concentration of solvent in the culture medium was maintained at 0.25% (v/v) to avoid solvent toxicity. Cells were incubated for 48 h in the absence or in the presence of extract. Cytotoxicity was assessed using the resazurin reduction test as described by O’Brien [23]) Fluorescence was measured on an automated 96-well Fluoroskan Ascent Fl plate reader (Labsystems) using an excitation wavelength of 530 nm and an emission wavelength of 590 nm. Cytotoxicity was expressed as the concentration of extract or compound inhibiting cell growth by 50% (IC_50_).
